# The efficiency of contact lens care regimens on protein removal from hydrogel and silicone hydrogel lenses

**Published:** 2010-01-20

**Authors:** Doerte Luensmann, Miriam Heynen, Lina Liu, Heather Sheardown, Lyndon Jones

**Affiliations:** 1 Centre for Contact Lens Research, School of Optometry, University of Waterloo, Waterloo, ON, Canada; 2 Department of Chemical Engineering, McMaster University, Hamilton, ON, Canada

## Abstract

**Purpose:**

To investigate the efficiency of lysozyme and albumin removal from silicone hydrogel and conventional contact lenses, using a polyhexamethylene biguanide multipurpose solution (MPS) in a soaking or rubbing/soaking application and a hydrogen peroxide system (H_2_O_2_).

**Methods:**

Etafilcon A, lotrafilcon B and balafilcon A materials were incubated in protein solutions for up to 14 days. Lenses were either placed in radiolabeled protein to quantify the amount deposited or in fluorescent-conjugated protein to identify its location, using confocal laser scanning microscopy (CLSM). Lenses were either rinsed with PBS or soaked overnight in H_2_O_2_ or MPS with and without lens rubbing.

**Results:**

After 14 days lysozyme was highest on etafilcon A (2,200 μg) >balafilcon A (50 µg) >lotrafilcon B (9.7 µg) and albumin was highest on balafilcon A (1.9 µg) =lotrafilcon B (1.8 µg) >etafilcon A (0.2 µg). Lysozyme removal was greatest for balafilcon A >etafilcon A >lotrafilcon B, with etafilcon A showing the most change in protein distribution. Albumin removal was highest from etafilcon A >balafilcon A >lotrafilcon B. H_2_O_2_ exhibited greater lysozyme removal from etafilcon A compared to both MPS procedures (p<0.001) but performed similarly for lotrafilcon B and balafilcon A lenses (p>0.62). Albumin removal was solely material specific, while all care regimens performed to a similar degree (p>0.69).

**Conclusions:**

Protein removal efficiency for the regimens evaluated depended on the lens material and protein type. Overall, lens rubbing with MPS before soaking did not reduce the protein content on the lenses compared to nonrubbed lenses (p=0.89).

## Introduction

The initial response of the immune system to isolate an implanted material from the body before fibrous or granulomatous tissue growths is the development of a coating consisting of a variety of proteins and lipids [1-3]. A similar response is found after a new contact lens is placed onto the ocular surface, with organic (proteins, mucins, and lipids) and inorganic (calcium, potassium, and chloride ions) tear-film elements in addition to exogenous components, such as cosmetics, forming a “coating” over the lens within minutes of exposure to the eye [4-10]. A variety of ocular complications during lens wear can be directly related to such deposition, particularly on soft contact lenses [11-16]. One particularly relevant complication is giant papillary conjunctivitis (GPC), which has been observed with a variety of materials and wearing schedules [14,15,17,18]. GPC has been closely linked with depositions of denatured proteins on the lens surface and potentially mechanical lens interactions with the under surface of the lids [11]. With modern lens materials, development of papillae on the palpebral conjunctiva is among the most prominent complications that occur during contact lens wear [19,20].

More than 100 different proteins have been identified in the human tear film [21,22], with a total concentration of 6.5–9.6 mg/ml [23]. This concentration may change over the day [24], during sleep [25], and under specific conditions, including stimulated tearing [26,27], increasing age [28], contact lens wear [29]], and in various eye diseases, such as Sjögren’s syndrome [30]. Lysozyme is of particular interest due to its high abundance and antimicrobial activity in the tear film [26,27,31]. It has a concentration in the tear film of 1.9 mg/ml [25,27], exhibits an overall positive charge with an isoelectric point pH of 11.1, and is constituted of 129 amino acids, which results in a molecular weight of 14.5 kDa [32]. Albumin is the most abundant protein in blood serum and is involved in the initial response to implanted biomaterials [2]. Albumin has 585 amino acids, a molecular weight of 66 kDa, and its concentration in the tear-film ranges from 0.02 to 0.04 mg/ml during the day [24,26] and rises to approximately 0.5 mg/ml after sleep [25,33,34]. Its overall negative charge (isoelectric point pH=4.7) results in a different sorption behavior compared to lysozyme [34-37]. However, both proteins have frequently been detected on ex vivo lenses [38-43].

Multipurpose care solutions (MPSs) and hydrogen peroxide-based systems (H_2_O_2_) are the most commonly used care regimens to clean and disinfect soft contact lenses [44]. Due to their convenience, MPS systems have become increasingly popular over the years and now account for approximately 90% of the market share for care regimens, with H_2_O_2_ being used by <10% of patients [44-46]. The majority of MPS systems were initially developed for use with conventional poly-2-hydroxyethylmethacrylate (pHEMA)-based materials and were prescribed using a manual rub and rinse step before overnight soaking of the lenses [47,48]. To improve convenience, several care systems were developed that were approved as “NO-RUB” products, with a brief rinse and long overnight soak only being required.

Silicone hydrogel (SH) contact lens materials provide high levels of oxygen to the cornea [49,50] and result in fewer hypoxic complications compared with conventional pHEMA-based materials [51,52]. The majority of SH materials are worn on a daily wear basis [53], and 90% of the patients wearing these materials on an overnight or continuous wear basis will remove the lenses at some point during the wearing cycle [54]. Once removed, the lenses require cleaning and disinfection before reinsertion.

Previous studies have reported that the deposition profile of SH and conventional pHEMA-based materials differ markedly, with SH materials depositing lower amounts of tear proteins, which are primarily denatured. On hydrogel biomaterials, denatured proteins are more tightly bound than native proteins [2,55], which raises the question of whether proteins bound to contact lens materials can be removed from the lens by rinsing and/or soaking alone.

Therefore, the purpose of this in vitro study was to investigate the efficiency of protein removal from pHEMA-based and SH contact lens materials, using commonly prescribed care regimens. The location and amount of two tear-film proteins (lysozyme and albumin) was determined before and after soaking the lenses, using either a polyhexamethylene biguanide-based MPS in a RUB or NO-RUB format or a NO-RUB H_2_O_2_ system.

## Methods

Two SH materials (lotrafilcon B, balafilcon A) and one pHEMA-based lens (etafilcon A) were investigated (Table 1). All lenses had a power of −3.0 diopters (D) and were presoaked in sterile phosphate buffered saline (PBS; 1.9 mM monobasic sodium phosphate, 8.1 mM dibasic sodium phosphate, 154 mM sodium chloride, pH 7.4) 24 h before protein incubation to remove any associated packaging components from the lens material.

**Table 1:** List of contact lenses investigated in this study.

**Trade name**	**USAN**	**FDA**	**Manufacturer**	**Surface modification**	**Water content (%)**	**Principal monomers**
ACUVUE® 2™	Etafilcon A	IV	Johnson & Johnson, Jacksonville, FL	none	58	HEMA, MAA
AIR OPTIX™ AQUA	Lotrafilcon B	I	CIBA Vision, Duluth, GA	25 nm high refractive index coating	33	DMA, TRIS, siloxane macromer
PureVision®	Balafilcon A	III	Bausch & Lomb, Rochester, NY	Plasma oxidation (glassy islands)	36	NVP, TPVC, NVA, PBVC

DMA *N,N*-dimethylacrylamide; HEMA 2-hydroxyethyl methacrylate; MAA methacrylic acid; NVA *N*-vinyl aminobutyric acid; NVP N-vinyl pyrrolidone; PBVC poly[dimethylsiloxyl] di [silylbutanol] bis[vinyl carbamate]; TPVC tris-(trimethylsiloxysilyl) propylvinyl carbamate; TRIS trimethyl siloxy silane.

Two techniques were used in this study to quantify and locate the protein of interest on the contact lens. In Experiment 1, a radiolabeling technique was used to quantify the overall amount of bound protein per lens, and in Experiment 2, confocal laser scanning microscopy (CLSM) identified the location of fluorescent-labeled protein on the surface and inside the lens matrix (bulk) (for conjugation methods, see below). Hen egg lysozyme (HEL; Sigma-Aldrich, St. Louis, MO) and bovine serum albumin (BSA; Sigma-Aldrich) were investigated in separate experiments, applying both the radiolabeling and CLSM method. BSA and HEL were substituted for human albumin and lysozyme primarily because of cost considerations; however, the shape and physicochemical properties between the proteins are similar, and they are expected to behave in an analogous manner [56-67].

**Figure 1:**
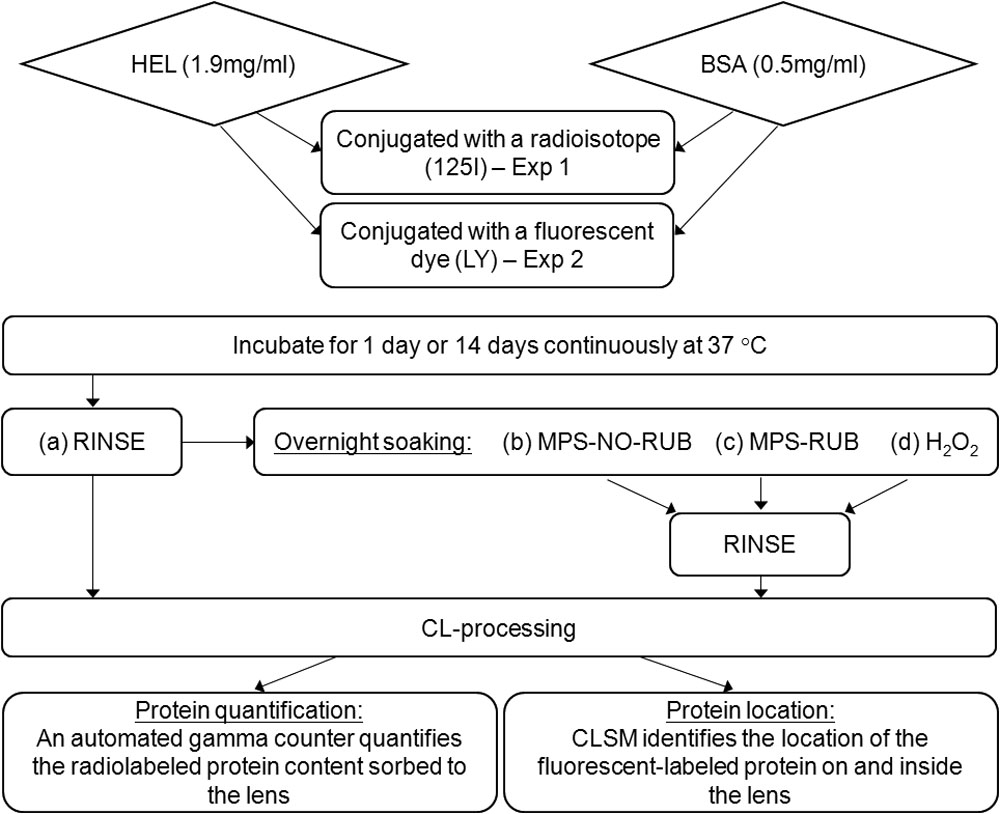
Schematic diagram for experimental procedures. Contact lenses were incubated in lysozyme (HEL) and albumin (BSA) solution, followed by overnight soaking in different care regimens and the two methods to locate and quantify the protein on the lens. In the figure, 125I indicates iodine^125^; BSA indicates bovine serum albumin; CL indicates contact lens; CLSM designates confocal laser scanning microscope; Exp indicates Experiment; H_2_O_2_ indicates hydrogen peroxide; HEL designates hen egg lysozyme; LY designates lucifer yellow vinyl sulfone; and MPS indicates multipurpose solution.

In both experiments lenses were incubated in amber glass vials filled with protein solution, with physiological concentrations of 1.9 mg/ml HEL [25] or 0.5 mg/ml BSA [34]. Etafilcon A is known to accumulate high levels of lysozyme [62,63] and was therefore incubated in 3 ml of HEL solution to ensure sufficient protein was available over the incubation period of 14 days. All other lens/protein combinations were soaked in 1 ml of solution. Three replicates were used for each condition, and the incubation was performed at 37 °C under constant rotation of 72 rpm for time periods of 1 and 14 days. Figure 1 provides an overview of the experimental procedures.

After protein incubation, lenses underwent one of four treatments, described below. The contact lens care regimens used in this study are listed in Table 2.

**Table 2:** List of care regimens used in this study.

**Trade name**	**Manufacturer**	**Disinfectant**	**Other constituents**	**Buffer**
ClearCare®	CIBA Vision, Duluth, GA	3% hydrogen peroxide	Pluronic 17R4	Phosphate
COMPLETE® MPS Easy Rub™	Abbot Medical Optics, Santa Ana, CA	Polyhexamethylene biguanide (0.0001%)	Poloxamer 237, Edetate disodium, Sodium chloride, Potassium chloride	Phosphate

• (a) “RINSE”—all lenses were held with plastic-tipped tweezers and gently swirled in 100 ml PBS for 3 s (repeated twice) and either processed immediately for protein localization or quantification or further prepared for overnight soaking using one of three differing treatments (b, c, or d).
• (b) “MPS-NO-RUB”—lenses were individually placed in COMPLETE® Easy Rub contact lens cases (Advanced Medical Optics, Santa Ana, CA), which were filled with 3 ml of COMPLETE® Easy Rub solution.
• (c) “MPS-RUB”—lenses were placed in a nitrile-gloved hand and 200 µl of COMPLETE® Easy Rub was added; using the index finger alone, five circular rotations on each side of the lens were performed (10 s total). The lenses were then briefly immersed in PBS before being placed in 3 ml of COMPLETE® Easy Rub solution.
• (d) “H_2_O_2_”—lenses were individually placed in ClearCare® lens cases (CIBA Vision, Duluth, GA), which were then filled with 9 ml of ClearCare® solution.

Lenses processed in treatments b, c, and d remained in the respective care solution overnight for 12 h, followed by immersing and swirling for 3 s in fresh PBS and further processing for protein localization or quantification (Figure 1).

Three replicates for each of the three lens types (etafilcon A, lotrafilcon B, and balafilcon A) were incubated in two proteins (HEL and BSA) at two time points (D1, D14), using four cleaning procedures (RINSE, MPS-NO-RUB, MPS-RUB, and H_2_O_2_) and two techniques to either quantify or locate the protein on the lens. This resulted in 288 lenses being examined.

### Experiment 1: Protein quantification using I^125^

In separate experiments, HEL and BSA were conjugated to I^125^ using the iodine monochloride method, as previously described [64,65]. Lenses were incubated in single protein solutions containing 1.9 mg/ml HEL or 0.5 mg/ml BSA, using a mixture of 2% labeled and 98% unlabeled protein (pH 7.4). Following the four treatments a, b, c, or d, the remaining protein content on the lens was determined by using an automated gamma counter (1470 Wallac Wizard; PerkinElmer, Woodbridge, ON). For quantification purposes, the radioactivity on each lens was converted into micrograms of protein [66,67].

### Experiment 2: Protein conjugation for confocal laser scanning microscopy

In separate labeling procedures, 180 mg HEL and BSA were dissolved in 0.05 M borate buffer (pH 8.5) and 0.04 M NaCl (HEL 5 mg/ml; BSA 10 mg/ml). The water soluble fluorescent dye Lucifer Yellow VS dilithium salt (LY; Sigma Aldrich) was dissolved in 1 ml of borate buffer (pH 8.5; 7 mg for BSA, 10 mg for HEL). The dye was added to the protein solution followed by gentle stirring for 1 h in the dark. Free LY was separated from the conjugated proteins using Sephadex G25 PD10 desalting columns (Amersham Biosciences, Piscataway, NJ). Following this, dialysis against PBS, using a 7-kDa molecular weight cutoff dialysis cassette, was performed until only negligible amounts of free LY were detected with a fluorescence spectrophotometer (F-4500; Hitachi, Tokyo, Japan). The labeling efficiency was calculated by determining the protein concentration in the solution, using the DC Protein Assay (Bio-Rad, Hercules, CA) and measuring the absorbance at 415 nm (which is the maximum absorbance for LY). The resulting degree of labeling was 0.26 for HEL and 2.94 for BSA (degree of labeling is the molecules of dye per molecule of protein).

### Contact lens incubation in fluorescent-labeled protein

The conjugated protein solutions were sterilized with 0.2 μm syringe filters (Pall Corporation, Ann Arbor, MI) to prevent microbial contamination of the samples during the incubation phase. Because lower amounts of labeled protein result in less photobleaching during subsequent laser scans and to allow consistent settings on the microscope throughout the experiment, the lowest possible ratio of conjugated HEL to unconjugated HEL was used. Contact lens materials that were known from previous studies [66,68] to accumulate large amounts of lysozyme were incubated with 2% labeled and 98% unlabeled HEL, while other materials known to accumulate only small amounts of protein were incubated in 100% labeled HEL. The final concentration of HEL was 1.9 mg/ml (pH 7.4). Because of the lower BSA sorption rate to contact lens materials [62], all lens types were incubated in 100% conjugated BSA.

### Confocal laser scanning microscopy examination technique

The center 4 mm of the lens was cut out using a mechanical punch press, and the sample was gently dabbed dry on lens paper before it was mounted onto a glass microscope slide. Approximately 40 μl of PBS was used as the mounting media. A glass coverslip was then carefully applied and sealed with nail polish to prevent evaporation and to stabilize the coverslip for use with the immersion objectives of the microscope.

The lens materials were subsequently examined for protein uptake by using CLSM (Zeiss Inc., Toronto, Canada). The Zeiss 510, configuration Meta 18, was equipped with an inverted motorized microscope Axiovert 200M. Each lens was scanned at four random locations by using an excitation wavelength of 405 nm (Laser Diode) and a long pass emission filter >505 nm. Each section of z stacks was set at 1-μm intervals, with image sizes of 512x512 pixels (230x230 µm). Lenses were scanned with a 40× water immersion C-Apochromat objective. Using the software provided with the microscope and ImageJ (U. S. National Institutes of Health, Bethesda, MD), the means of the fluorescence intensity were plotted as a function of the scanning depth. In preliminary tests optimized settings for laser intensity, detector gain, and amplifier offset were determined using new lenses as controls that were presoaked in PBS. This was necessary to reduce the impact of background noise from the various lens materials. These CLSM scan settings remained unchanged throughout the entire experiment.

For statistical analysis of the quantitative protein uptake and protein location, repeated measures analysis of variance (ANOVA) was applied, followed by post hoc comparisons using Tukey's honestly significant difference test. A p<0.05 was considered significant. To determine the significance of differences between the amount of protein sorbed to the investigated materials, a comparison between the RINSE data was tested using the factors “Protein” (HEL and BSA), “Material” (etafilcon A, lotrafilcon B, and balafilcon A), and “Time” (D1 and D14). The cleaning efficiency was analyzed individually for each lens–protein combination because of the wide range of protein uptake between lens materials. Differences between the amounts of protein were determined separately for each lens material (etafilcon A, lotrafilcon B, and balafilcon A) with the two factors “Time” (D1 and D14) and “Treatment” (RINSE, MPS-NO-RUB, MPS-RUB, and H_2_O_2_), including interactions.

To determine differences in protein location, each CLSM lens scan was sectioned into front and back surface and “bulk” regions, as previously described [69]. Briefly, the fluorescence intensity on the front and back surface was calculated by averaging the five micron scan steps around the front and back “surface peak” and the “bulk” intensity was calculated by averaging the innermost 30 µm of the lens scan [69]. As noted in our previous study [69], the relative fluorescence signal on the back surface typically showed a minor decrease compared to the front surface. This is due to increased absorbance of the laser light when measuring deeper into the lens material and could be seen in the majority of cases. Therefore, comparisons between protein location on and within the lens focused only on differences in the front surface versus the bulk (central) region, with the assumption that both surfaces accumulated similar amounts of protein. The scaling of the CLSM results was based on arbitrary units and solely allowed comparisons between a single protein type on one specific material. Using repeated measures ANOVA, significant differences in fluorescence intensity were determined separately for each lens material (etafilcon A, lotrafilcon B, and balafilcon A), with the three main effects “Time” (D1 and D14), “Treatment” (RINSE, MPS-NO-RUB, MPS-RUB, and H_2_O_2_), and “Location” (front surface, back surface, and bulk), including interactions. The fluorescence signal did not provide quantitative results and the units cannot be compared directly between materials, therefore radiolabeled protein was used for quantitative comparisons.

## Results

Etafilcon A, lotrafilcon B, and balafilcon A incubated in I^125^-labeled protein showed significantly more HEL on all lens types compared to BSA at all time points (p<0.001). An increase in HEL and BSA sorption was found on all three lens materials over time (p<0.05), except for BSA in combination with etafilcon A (p=0.48).

Following incubation of the three contact lens materials in either 1.9 mg/ml HEL or 0.5 mg/ml BSA, the total amount and location of protein on these materials was determined before and after overnight soaking in MPS with and without manual lens rubbing (MPS-RUB, MPS-NO-RUB) or H_2_O_2_.

Etafilcon A accumulated the highest amounts of HEL (mean 2,200 µg/lens) and the lowest amounts of BSA (mean 0.2 µg/lens) compared to the other materials (p<0.001) (Table 3). After overnight soaking, both care regimens removed significant amounts of both proteins from this lens type (p<0.001). After 14 days of incubation, H_2_O_2_ removed 24.3% of the HEL from etafilcon A, which was significantly more (p<0.001) compared to MPS-RUB (15.8%) and MPS-NO-RUB (16.3%), which were not significantly different to each other (p=0.88). The low amounts of BSA were significantly reduced (p<0.001) by 62.4%, 62.2%, and 55.5% by using H_2_O_2_, MPS-RUB, and MPS-NO-RUB, respectively, with all cleaning procedures performing similarly (p>0.98; Table 3).

**Table 3:** Total amount of hen egg lysozyme and bovine serum albumin sorbed to etafilcon A after 1 and 14 days of incubation, followed by the treatments RINSE, MPS-NO-RUB, MPS-RUB, or H_2_O_2_ (Mean±95% confidence interval).

**ETAFILCON A**	**HEL**			**BSA**		
	**D1**	**D14**	Difference between treatments*	**D1**	**D14**	Difference between treatments*
RINSE (a)	1139.8±7.14	2200.3±15.64	(b), (c), (d)	0.16±0.037	0.20±0.037	(b), (c), (d)
MPS-NO-RUB (b)	909.3±5.52	1852.1±19.16	(a), (c), (d)	0.03±0.006	0.09±0.005	(a)
MPS-RUB (c)	906.6±13.00	1841.5±10.38	(a), (b)	0.02±0.004	0.08±0.013	(a)
H_2_O_2_ (d)	783.4±11.13	1666.1±15.83	(a), (b)	0.02±0.004	0.08±0.041	(a)

*Overall differences between treatments (p<0.05), using the combined time points.

**Figure 2:**
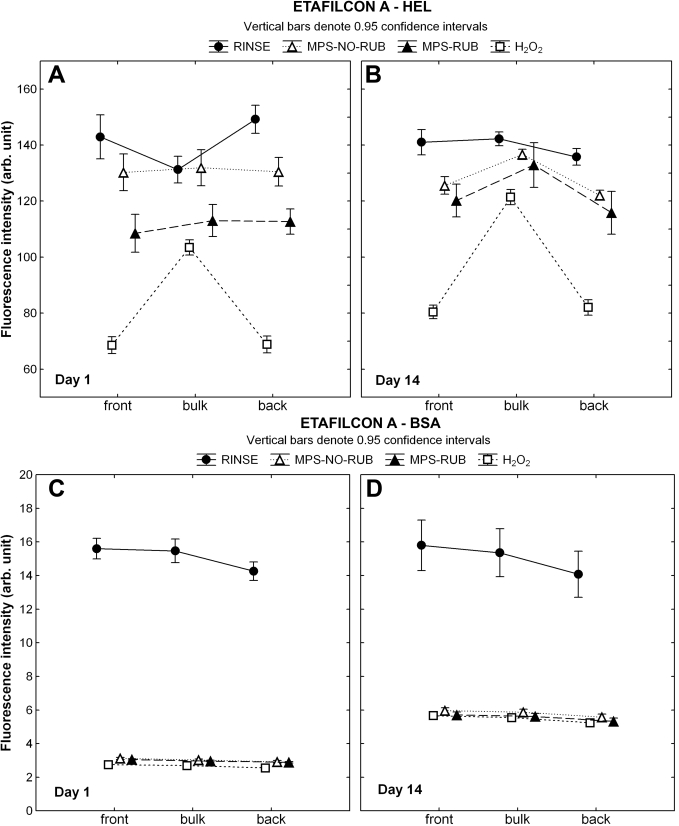
Lysozyme and albumin distribution through etafilcon A. CLSM (confocal laser scanning microscopy) scans were analyzed to locate the fluorescent-conjugated protein on the front surface, within the bulk region, and on the back surface of etafilcon A after one and 14 days of incubation. Lenses were either rinsed in phosphate buffered saline (PBS), soaked overnight in hydrogen peroxide (H_2_O_2_) or soaked overnight in a multipurpose solution (MPS) with (MPS-RUB) or without (MPS-NO-RUB) manual lens rubbing. **A**, **B**: These panels show the results for HEL (hen egg lysozyme), **C**, **D**: These panels show the results for BSA (bovine serum albumin).

The CLSM results for the different cleaning treatments show the distribution of the fluorescent-conjugated protein on the front surface, within the central lens bulk, and at the back surface of etafilcon A (Figure 2A–D). Following the RINSE procedure alone, HEL sorption to etafilcon A showed a slightly higher protein density on the surface compared to the bulk region on D1 (p<0.001), but this leveled out over time, with no difference being seen on D14 (p=1.0). For both time points, soaking in H_2_O_2_ removed significantly higher amounts of HEL from the surface of etafilcon A compared to all the other procedures (p<0.001), and significantly more HEL was measured in the central region than on the surface (p<0.001). This phenomenon was seen to this extent only with this specific lens protein care regimen combination. For the MPS on D1, both techniques removed significant amounts of protein from both the surface and bulk regions compared with RINSE alone (p<0.001). No differences between the surface and bulk regions were measured for either RUB or NO-RUB methods, but there was a reduction in both regions on D1 when the lens was rubbed (p<0.001). Both techniques, RUB and NO-RUB, removed more HEL from the surface than from the bulk region on D14, but there was no significant difference between the two techniques (p=0.64).

The overall BSA sorption to etafilcon A with the RINSE procedure was similar at both time points (p=1.00), showing an almost even distribution of the protein at the surface and in the bulk region, as seen in Figure 2C,D. The use of MPS and H_2_O_2_ showed a successful removal of BSA from both the surface and the bulk regions (p<0.001), with a slightly reduced efficiency on D14. There were no significant differences between the three procedures for either time points (p>0.95).

Lotrafilcon B accumulated higher quantities of HEL (mean 9.65 µg/lens) compared to BSA (mean 1.82 µg/lens) after 14 days of incubation in radiolabeled protein solution (p<0.001; Table 4). Following overnight soaking none of the care regimens removed appreciable amounts of HEL from this lens type (p>0.46), while a small but statistically significant reduction was seen for BSA when exposed to H_2_O_2_ (p<0.049). After 14 days of incubation, H_2_O_2_ removed 7.2% HEL from lotrafilcon B, which was similar to both MPS-RUB (3.6%) and MPS-NO-RUB (2.9%) (p>0.90). The amount of BSA removed was slightly more (14.0%, 11.9%, and 11.0%) using H_2_O_2_, MPS-RUB, and MPS-NO-RUB, respectively, with all cleaning procedures performing similarly (p>0.89; Table 4).

**Table 4:** Total amount of hen egg lysozyme and bovine serum albumin sorbed to lotrafilcon B after 1 and 14 days of incubation, followed by the treatments RINSE, MPS-NO-RUB, MPS-RUB, or H_2_O_2_ (Mean±95% confidence interval).

**LOTRAFILCON B**	**HEL**			**BSA**		
	**D1**	**D14**	Difference between treatments*	**D1**	**D14**	Difference between treatments*
RINSE (a)	5.14±0.64	9.65±1.54	-	0.84±0.10	1.82±0.19	(d)
MPS-NO-RUB (b)	3.28±0.85	9.37±1.72	-	0.54±0.07	1.62±0.08	-
MPS-RUB (c)	3.98±0.43	9.31±2.09	-	0.46±0.19	1.60±0.25	-
H_2_O_2_ (d)	4.41±0.84	8.96±1.28	-	0.45±0.05	1.57±0.13	(a)

*Overall differences between treatments (p<0.05), using the combined time points.

**Figure 3:**
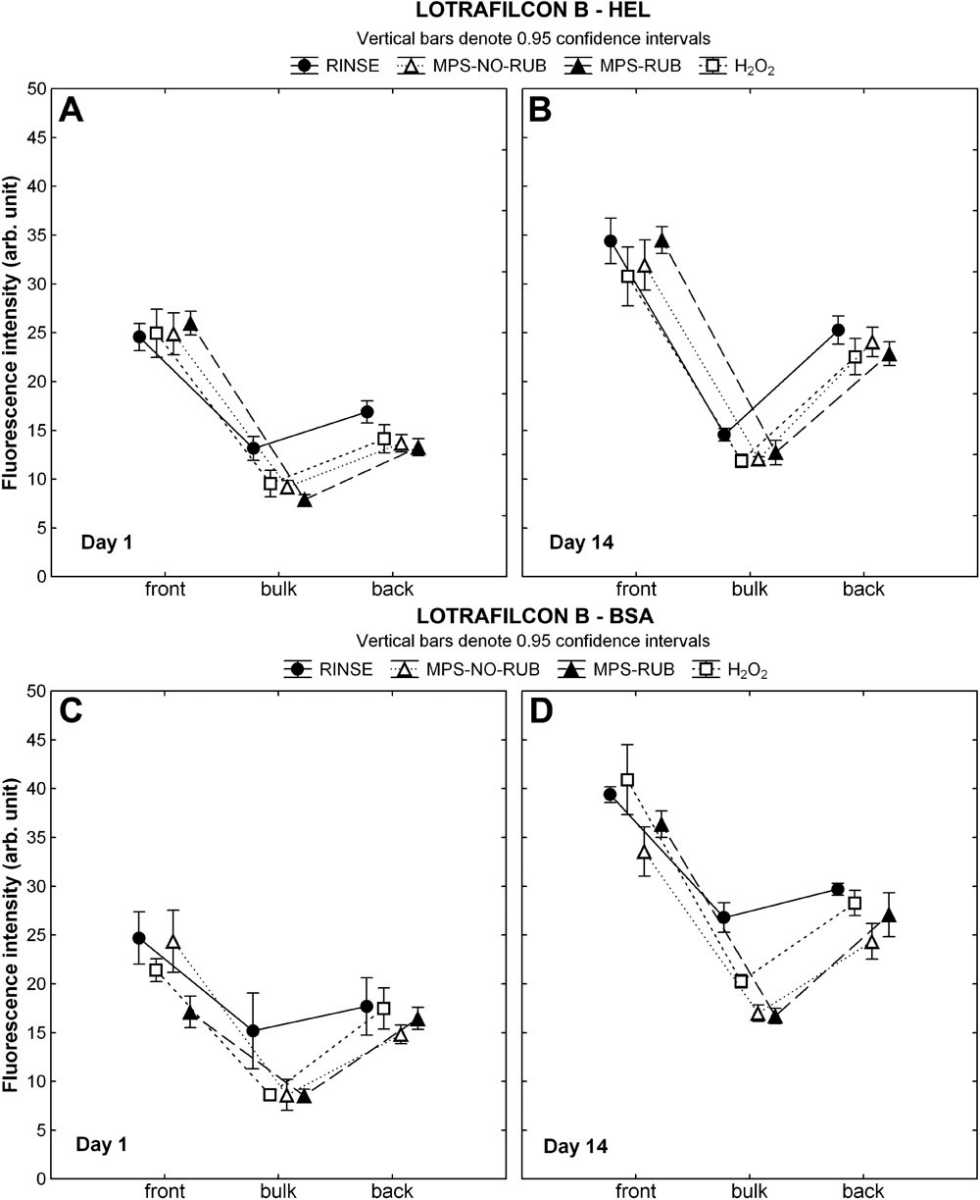
Lysozyme and albumin distribution through lotrafilcon B. CLSM (confocal laser scanning microscopy) scans were analyzed to locate the fluorescent-conjugated protein on the front surface, within the bulk region, and on the back surface of lotrafilcon B after one and 14 days of incubation. Lenses were either rinsed in phosphate buffered saline (PBS), soaked overnight in hydrogen peroxide (H_2_O_2_) or soaked overnight in a multipurpose solution (MPS) with (MPS-RUB) or without (MPS-NO-RUB) manual lens rubbing. **A**, **B**: These panels show the results for HEL (hen egg lysozyme), **C**, **D**: These panels show the results for BSA (bovine serum albumin).

The location of fluorescent-conjugated HEL on the surface and within the bulk region of lotrafilcon B is shown in Figure 3A,B. Significantly higher amounts of HEL were detectable on the surface of lotrafilcon B following the RINSE procedure compared to the bulk region on D1, which became even more distinct on D14 (p<0.001). Overnight soaking in H_2_O_2_ or MPS with or without rubbing removed protein solely from the central lens region on D1 (p<0.04); however, the front surface on D1 and both locations on D14 did not show a significant decrease in protein accumulation using any of the three procedures (p>0.3), with the exception of the surface on D14 after soaking in H_2_O_2_ (p=0.03).

BSA sorption to lotrafilcon B showed a trend similar to HEL, with more BSA detected on the surface compared to the bulk region after the RINSE procedure, as seen in Figure 3C,D (p<0.001). All cleaning techniques removed BSA from the central location at both time points (p<0.001). For the surface, only MPS-RUB removed significant amounts of BSA on D1 and only MPS-NO-RUB reduced the BSA content on D14 (p<0.001). On D14, both MPS applications removed more protein from the surface compared to the H_2_O_2_ care solution (p<0.01), but no differences could be detected for the bulk region (p>0.05). Although MPS-RUB removed more BSA from the surface on D1 compared to the NO-RUB technique, all other locations were not different on either time points for the two MPS procedures (p>0.35).

Balafilcon A accumulated much higher amounts of HEL (mean 50.0 µg/lens) compared to BSA (mean 1.90 µg/lens) after 14 days of incubation in I^125^-conjugated protein (p<0.001; Table 5). After overnight soaking both care regimens removed significant amounts of both proteins from this lens type (p<0.01). After 14 days of incubation, HEL was more efficiently removed from balafilcon A compared to the other two lens materials, with similar proportions of 59.9%, 58.4%, and 61.4% for H_2_O_2_, MPS-RUB, and MPS-NO-RUB, respectively (p<0.001). For BSA, H_2_O_2_ removed 31.7%, which was similar to MPS-RUB (30.7%) and MPS-NO-RUB (29.2%). The three cleaning procedures showed overall similar protein removal efficiencies for both BSA and HEL (p>0.69; Table 5).

**Table 5:** Total amount of hen egg lysozyme and bovine serum albumin sorbed to balafilcon A after 1 and 14 days of incubation, followed by the treatments RINSE, MPS-NO-RUB, MPS-RUB, or H_2_O_2_ (Mean±95% confidence interval).

**BALAFILCON A**	**HEL**			**BSA**		
	**D1**	**D14**	Difference between treatments*	**D1**	**D14**	Difference between treatments*
RINSE (a)	42.71±1.93	50.00±0.14	(b), (c), (d)	0.63±0.08	1.90±0.37	(b), (c), (d)
MPS-NO-RUB (b)	8.08±0.39	19.31±0.99	(a)	0.24±0.05	1.35±0.31	(a)
MPS-RUB (c)	7.69±0.71	20.80±3.30	(a)	0.20±0.04	1.32±0.30	(a)
H_2_O_2_ (d)	8.18±0.47	20.08±1.69	(a)	0.21±0.03	1.30±0.31	(a)

*Overall differences between treatments (p<0.05), using the combined time points.

**Figure 4:**
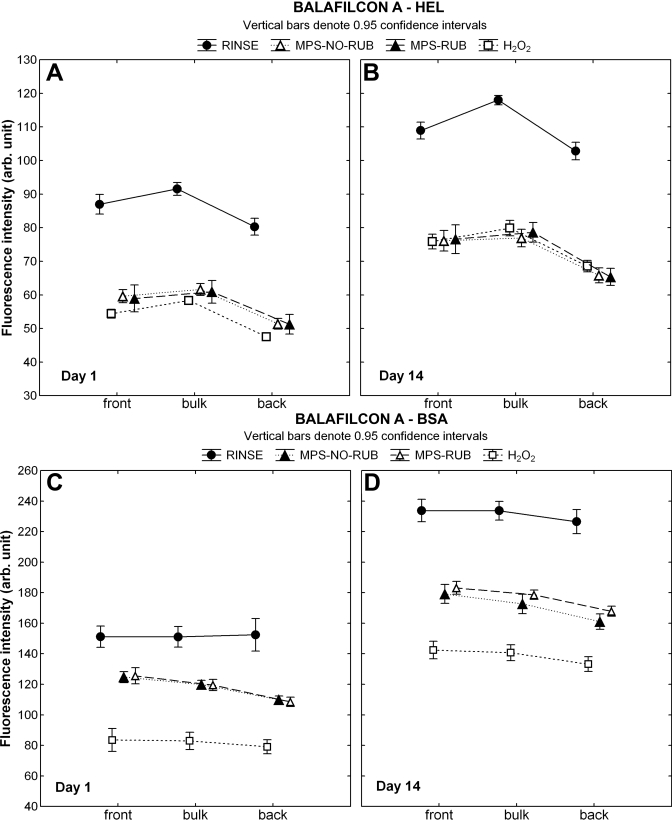
Lysozyme and albumin distribution through balafilcon A. CLSM (confocal laser scanning microscopy) scans were analyzed to locate the fluorescent-conjugated protein on the front surface, within the bulk region, and on the back surface of balafilcon A after one and 14 days of incubation. Lenses were either rinsed in phosphate buffered saline (PBS), soaked overnight in hydrogen peroxide (H_2_O_2_) or soaked overnight in a multipurpose solution (MPS) with (MPS-RUB) or without (MPS-NO-RUB) manual lens rubbing. **A**, **B**: These panels show the results for HEL (hen egg lysozyme), **C**, **D**: These panels show the results for BSA (bovine serum albumin).

Imaging results of the fluorescent-conjugated protein indicated a higher HEL density inside the bulk region compared to the surface of balafilcon A following the RINSE procedure at both time points (p<0.001; Figure 4A,B). All three cleaning techniques removed significant amounts of HEL from both the surface and bulk region (p<0.001). Soaking in H_2_O_2_ removed more HEL from the surface of balafilcon A on D1 compared to the MPS applications (p<0.02); however, on D14 all care regimens performed similarly for both surface and bulk regions (p>0.09).

BSA showed an equal distribution throughout the balafilcon A material at both time points following the RINSE procedure (p=1.0), as shown in Figure 4C,D. All cleaning procedures removed significant amounts of BSA from the surface and the bulk material (p<0.001). At both time points a higher protein reduction was seen when using H_2_O_2_ compared to both MPS applications (p<0.001), which were not different to each other on D1 (p=1.0). Small but significant differences could be seen on the lens surface and within the bulk region between both MPS procedures on D14, with MPS-NO-RUB removing more BSA compared to MPS-RUB (p<0.05).

## Discussion

Current soft contact lens care regimens have been evaluated for their efficiency against both microbial and tear-film deposition on various soft lens materials [48,70-77]. Both ex vivo and in vitro studies have demonstrated material-specific sorption profiles and have confirmed differences between care regimens for removing non-pathogenic (e.g., lipids, proteins) [48,70,74-77] and microbial (e.g., bacteria, fungi) [70-73] components from the lens. Furthermore, manual lens rubbing reduces the appearance of visual deposition by removing general tear-film components and cosmetics from the lens more effectively compared with soaking alone [78,79]. A clinical study conducted by Nichols [78] determined visual deposition on patient-worn SH lenses after using various MPSs in a rub and no-rub application. The subjective grading method demonstrated an overall reduction in lens “haze” for manually rubbed lenses that were cleaned using either COMPLETE® MoisturePLUS or Alcon Opti-Free Express. Cho et al. [79] reported similar results from an in vitro study investigating ionic high-water pHEMA-based lens materials that were artificially deposited with albumin, hand cream, and mascara. Lenses that were not rubbed before the soaking process showed similar levels of deposition regardless of the rinsing duration. In contrast, all four MPS systems investigated removed significant amounts following extensive lens rubbing [79].

While both of the above studies clearly describe differences between care regimens and their method of use, it still remains unclear if the deposited species were removed primarily from the lens surface or also from the central or bulk lens region. Of particular interest to us was the impact of care regimens and rubbing on the removal of tear-film proteins and whether such proteins are removed differentially from the surface or bulk locations. Therefore, the purpose of this study was to determine the efficiency of various contact lens care regimens on the removal of two typical tear-film proteins, lysozyme (HEL) and albumin (BSA), which differ markedly in size, charge, and concentration.

As shown in previous studies [67,69], the protein distribution profile for BSA and HEL differs significantly between lens materials. The pHEMA material etafilcon A (Food and Drug Administration [FDA] group IV) allowed both proteins to penetrate the lens matrix, while the high refractive index coating and/or the properties of the lotrafilcon B SH bulk material [80] (FDA group I) minimized protein penetration into the material, with both proteins primarily being deposited on the surface region [67,69]. It may be assumed that protein sorbed onto the lens surface would be easier to remove than protein penetrating the lens matrix. However, results from the current study showed no change in the overall HEL amount (by radiolabeling) and distribution profile (by CLSM imaging) on the lotrafilcon B material after overnight soaking using a RUB or NO-RUB application (Table 4 and Figure 3A,B). BSA amounts were slightly reduced (by 11–14%) for this material by either lens rubbing or using H_2_O_2_ systems (Table 4), and the CLSM results confirmed that BSA was removed primarily from the bulk region and not from the surface region (Figure 3C,D).

Etafilcon A allowed both BSA and HEL to fully penetrate the lens bulk region over time [67,69], and our study demonstrated that using either MPS or H_2_O_2_ removed significant amounts of lysozyme (15.8–24.3%) and BSA (55.5–62.4%) from both the lens surface and bulk regions (Table 3 and Figure 2A-D). When examining the differences between these regimens, it is clear that H_2_O_2_ removed substantially more HEL from the surface region of etafilcon A than either MPS method (Table 3 and Figure 2A,B). This phenomenon was not observed with BSA, which deposited substantially less than HEL (Table 3). The high levels of deposition of the positively charged HEL on ionically charged materials, such as etafilcon A, has been shown previously [37,66,81].

Our results suggest that both proteins were less tightly bound when sorbing to the surface of etafilcon A (Figure 2A-D) compared to the lotrafilcon B material (Figures 3A-D), which showed no (or minimal) protein removal from the surface following any of the cleaning procedures. These findings were also confirmed in the quantitative results, which showed a higher percentage of protein removal for etafilcon A compared to lotrafilcon B (Figure 2A,B) This may be due to the conformational state of the proteins, which were sorbed to the more hydrophilic surface (etafilcon A) compared to the more hydrophobic surface (lotrafilcon B) typically exhibited by SH materials [82]. Previous studies have determined changes in the secondary structure for HEL and BSA when depositing on contact lenses and have shown a higher denaturation rate for proteins sorbed to SH materials compared to pHEMA materials [62,63,68,75,83]. Furthermore, denatured proteins typically bind more tightly to surfaces compared to native proteins [3,55,62], which may explain our difficulties in removing either protein from lotrafilcon B. Thus, our data suggest that when HEL deposits on lotrafilcon B, it is difficult to remove, regardless of the care regimen employed. In comparison BSA sorbs to a lesser extent and is marginally easier to remove.

The balafilcon A material is surface modified using a plasma oxidation method, which results in hydrophilic silicate islands distributed over the lens surface [84]. This study showed that this surface modification procedure was no barrier for either protein as they both fully penetrated the entire matrix (Figure 4A-D). In comparison to the increased surface build up of both proteins seen on lotrafilcon B, balafilcon A accumulated slightly more HEL in the lens bulk compared to the surface (Figure 4A,B) but showed an almost even distribution for BSA (Figure 4C,D). The highly porous and hydrophilic structure of balafilcon A [85-87] allowed easy ingress of both proteins, particularly the smaller HEL, and appeared to allow relatively easy removal of either protein deposited, using any of the care regimens investigated, on both the surface and from within the bulk region. Our quantitative experiment showed the highest HEL reductions on this material, with 58.4–61.4% removal after overnight soaking using any of the three treatments (Table 5), which was similarly reflected by the CLSM imaging data showing an equal reduction throughout the balafilcon A material (Figure 4A,B). The results for BSA show removal efficiencies of 29.2–31.7%, and although the results were slightly higher for H_2_O_2_, significant differences were only seen with the fluorescence imaging technique (Figure 3C,D).

The amount of protein removal from the lens materials investigated in this study can be compared to previous findings from both Franklin [47] and Jung [77] who investigated pHEMA-based materials only. Franklin incubated FDA groups I, II, and III in an artificial tear solution containing various proteins and lipids. Lenses were manually rubbed with various single and multipurpose solutions, and the protein content was determined using fluorescence spectroscopy. Franklin reported a protein reduction of 27–45% for MPS care regimens [47], which is in close agreement with findings from Jung et al. [77] who reported protein removal efficiencies of 28–52% using H_2_O_2_ and MPS regimens. In Jung’s in vitro study [77], which examined FDA groups I–IV, proteins were extracted and quantified using a protein assay. The results for FDA group IV lenses showed a more efficient protein removal using H_2_O_2_ compared to the polyhexamethylene biguanide-based MPS system, which is in agreement to the results from our study using etafilcon A as our FDA group IV lens.

The protein removal efficiency in our study, as evidenced by the radiolabeled results, ranged from 2.9% to 62.4%, which suggests that not only do care regimens impact the removal efficiency but that this removal is also markedly influenced by the specific characteristics of the lens materials investigated.

As described above two recent studies have shown that rubbing lenses reduces visible deposition in both in vitro and in vivo studies [78,79]. In our study, relatively minor differences between deposition of two common tear-film proteins were demonstrated using an MPS system in a RUB or NO-RUB format. Potential reasons why our in vitro study was not able to mimic previous results is that this is the only study to date to quantify protein removal from SH materials by using RUB versus NO-RUB methods. The Nichols’ paper [78] examined the removal of visible tear-film deposits from a single SH material (galyfilcon A), which was not examined in our study. The study by Cho and colleagues [79] examined visible deposition, including albumin, but used an FDA group IV material (ocufilcon D) that was also not examined in our study. One potential issue to consider relative to patient use of such systems is that our laboratory-based experiment used nitrile-gloved hands for the RUB technique. Although the gloves had textured finger tips to improve grip, potential differences to ungloved hands may occur. Another limitation of our in vitro experiment is that the lenses were incubated in the protein solution alone, which does not provide the intermittent surface drying that occurs between blinks in in vivo studies and which may have impacted on the deposition results. In reality, a follow-up ex vivo study in which lenses are harvested and examined for deposited proteins from a clinical study in which human subjects use an MPS in a RUB and then NO-RUB format is required to unequivocally demonstrate differences between these formats. Although the differences in protein removal between our two techniques using MPS in a RUB and NO-RUB application are minor, it must be considered that this may be entirely different for the removal of lipids, microorganisms [88,89], and other debris.

The use of lysozyme and albumin in single protein solutions describes the interaction between the protein and material of interest; however, this sorption behavior may change with the addition of an artificial tear solution, which includes more proteins, lipids, mucins, and ions. A competitive process of protein adsorption and desorption is expected as smaller proteins get replaced by proteins with higher surface affinity [43].

Biocompatible materials tend to bind proteins relatively loosely, and these proteins are often easy to remove as they maintain their conformation. This compares with “less biocompatible” surfaces, which are typically more hydrophobic and have a tendency to denature proteins over time, potentially stimulating inflammatory responses in the biologic host [62,90-96]. Future work within the contact lens arena should focus on the development of surfaces that maintain protein activity and allow for easier removal of deposited tear-film components, possibly by developing contact lens care regimens and SH materials that are optimized to work together.

In conclusion, the efficiency of protein removal varied greatly between contact lens materials, care regimens, and proteins investigated. MPS in a RUB and NO-RUB application and H_2_O_2_ removed significantly higher amounts of HEL and BSA from etafilcon A and balafilcon A compared to lotrafilcon B. Comparisons between care regimens showed a slightly better efficiency for the H_2_O_2_ system compared to MPS-RUB or NO-RUB, which showed negligible differences between these preserved systems.
